# Sterile Alpha Motif Domain-Containing 5 Suppresses Malignant Phenotypes and Tumor Growth in Breast Cancer: Regulation of Polo-Like Kinase 1 and c-Myc Signaling in a Xenograft Model

**DOI:** 10.7759/cureus.73259

**Published:** 2024-11-07

**Authors:** YouLin Tuo, YiFeng Ye

**Affiliations:** 1 Department of Breast Surgery, Sichuan Provincial People's Hospital, University of Electronic Science and Technology of China, Chengdu, CHN

**Keywords:** breast cancer, plk1, samd5, the c-myc signaling, triple-negative breast cancer

## Abstract

Background

Breast cancer, particularly the triple-negative breast cancer (TNBC) subtype, remains a significant clinical challenge due to its resistance to standard chemotherapy and high recurrence rate. In this study, we explored the role of Sterile Alpha Motif Domain-Containing 5 (SAMD5) as a potential regulatory partner with the c-Myc oncogenic signaling pathway in breast cancer.

Materials and methods

Functional assays were conducted to investigate the effects of SAMD5 overexpression on cell viability, colony formation, and invasive behavior in TNBC cell lines. This study further assessed the expression levels of proliferation and invasion markers, including Ki67 (a marker for cell proliferation), Matrix Metalloproteinase-2 (MMP2), and Matrix Metalloproteinase-9 (MMP9). Mechanistic analyses identified a negative correlation between SAMD5 and Polo-like Kinase 1 (PLK1), a gene frequently overexpressed in breast cancer, particularly in TNBC. The effects of PLK1 knockdown on cell viability, colony formation, and invasion were observed, along with the impact of PLK1 overexpression on SAMD5’s inhibitory activity. In vivo studies were performed using a xenograft tumor model in nude mice to evaluate the impact of SAMD5 overexpression on tumor weight and volume.

Results

SAMD5 overexpression significantly reduced cell viability, colony formation, and invasion in TNBC cells, and downregulated key proteins in the c-Myc signaling pathway, including c-Myc itself, β-catenin, Cyclin-Dependent Kinase 4 (CDK4), Cyclin-Dependent Kinase 6 (CDK6), and Cyclin D1. PLK1 overexpression was found to counteract SAMD5’s inhibitory effects. In vivo experiments demonstrated that SAMD5 overexpression led to a marked reduction in tumor weight and volume, effects that were partially reversed by PLK1 overexpression.

Conclusions

SAMD5 acts as a tumor suppressor in breast cancer, particularly in TNBC, by inhibiting critical cellular processes and downregulating the c-Myc signaling pathway. This effect appears to be mediated, in part, through its negative association with PLK1. Targeting the SAMD5/PLK1 axis offers a promising therapeutic strategy for addressing aggressive breast cancers.

## Introduction

Breast cancer is the most common malignant tumor in women and the second leading cause of cancer death globally [1]. Based on molecular markers such as the estrogen receptor (ER), progesterone receptor (PR), and human epidermal growth factor receptor 2 (HER2), breast cancer can be classified into three main subtypes: hormone receptor (HR)-positive, HER2-positive, and triple-negative breast cancer (TNBC). TNBC, accounting for about 15-20% of all breast cancers [2], is associated with a poorer prognosis compared to HR-positive subtypes, with more than 50% of patients relapsing within three to five years post-diagnosis [3] and a median overall survival of 10.2 months with current treatments [4]. The absence of ER, PR, and HER2 markers in TNBC renders endocrine and HER2-targeted therapies ineffective, leaving nonspecific chemotherapy as the standard treatment [5]. Although TNBC shows the highest response to conventional chemotherapy strategies, including taxanes and anthracyclines, fewer than 30% of patients achieve a full response, and recurrence and death rates remain elevated compared to non-TNBC subtypes [2]. Hence, there is an urgent need to identify novel prognostic biomarkers and therapeutic targets to enhance treatment efficacy and patient outcomes in breast cancer, particularly in TNBC.

The c-Myc gene is located on human chromosome 8, encoding the transcription factor c-Myc and playing a pivotal role in cell cycle progression, proliferation, apoptosis, and cellular transformation [6,7]. Regulation of c-Myc expression involves multiple mechanisms at the transcriptional level, particularly through the proximal promoter region [8]. c-Myc serves as a transcription factor and forms a heterodimer with Myc-associated factor X (MAX), enabling it to bind to DNA and regulate gene expression [9,10]. In breast cancer, c-Myc is frequently overexpressed, contributing significantly to tumor development and progression [11-13]. Emerging evidence highlights c-Myc's critical effect on regulating the tumor microenvironment (TME), which includes multiple cell types, such as cancer-associated fibroblasts (CAFs), tumor-associated macrophages (TAMs), and vascular endothelial cells (VECs), among others [14-17]. Non-cellular components like the extracellular matrix (ECM) and soluble signaling molecules also play essential roles in TME dynamics [18,19]. Given the multiple effects of c-Myc signaling on breast carcinoma, identifying factors related to or regulating c-Myc signaling might provide new therapeutic targets and improve treatment outcomes.

Herein, using online datasets, differentially expressed genes (DEGs) in breast cancer samples were identified, and Sterile Alpha Motif Domain-Containing 5 (SAMD5) was found. SAMD5 is mainly negatively associated with cell cycle proliferation and other pathways [20,21], in which the related genes show highly significant negative correlation enrichment on HALLMARK_MYC_TARGETS_V1/V2. Therefore, we hypothesized that SAMD5 might be related to the Myc pathway. The expression and functions of SAMD5 in breast cancer cells were investigated. Factors that might interact with SAMD5 were analyzed, and Polo-like Kinase 1 (PLK1) was identified. The expression and functions of PLK1 in breast cancer cells were investigated. SAMD5 regulation of PLK1 was validated, and the dynamic effects of SAMD5/PLK1 on breast cancer cell phenotypes, c-Myc signaling, and in vivo tumor growth in a mouse model were explored.

## Materials and methods

Differential gene expression analysis

The Cancer Genome Atlas-Breast Invasive Cancer (TCGA-BRCA) expression data were downloaded from Xena (https://xenabrowser.net/datapages/), generated using the IlluminaHiSeq platform, which includes 114 normal tissue samples and 1,104 breast cancer samples of various types. The limma package was applied to analyze DEGs. The breast cancer expression datasets GSE57297 and GSE22820 were used for further screening of DEGs. The limma package was employed for the analysis. The DEGs identified were compared to determine any overlapping genes. GSE70947, GSE57297, GSE38959, GSE33447, GSE7904, GSE22820, GSE10780, GSE21442, and GSE21444 were used for expression validation.

Clinical relevance analysis

Kaplan-Meier Plotter (KMplot) online tools (https://www.kmplot.com/analysis/) were utilized to analyze the prognostic value of SAMD5 expression in patients with breast cancer, according to gene chip and RNA-seq data of breast cancer in KMplot. The expression of SAMD5 across different pathological stages and TNM classifications was evaluated using the TCGA-BRCA data and clinical features. TIMER (http://timer.comp-genomics.org/) was employed to analyze the association between SAMD5 expression and immune cell infiltration, as well as tumor purity.

Pathway enrichment analysis

The TCGA-BRCA dataset was used for Pearson correlation analysis of SAMD5 expression with the expression of 20,530 genes to identify significant pathways.

Clinical tissue sample collection

Human tissue-based studies were approved by the Ethics Committee of the Sichuan Academy of Medical Sciences and Sichuan Provincial People’s Hospital, Chengdu, China (approval no. TYL20210817). A total of 12 breast cancer tissues and paired non-tumor adjacent tissues were obtained from patients undergoing surgery at the Sichuan Academy of Medical Sciences and Sichuan Provincial People’s Hospital, University of Electronic Science and Technology of China, all of whom provided written consent. The tissues were fixed in formalin for histological analysis or stored at -80℃ for RNA isolation.

Histopathological examination

After being formalin-fixed and paraffin-embedded, tumor tissues were subjected to histopathological examination. From these paraffin blocks, tissues were sectioned into slices at a thickness of 4 μm, followed by hematoxylin and eosin (H&E) staining. Next, a light microscope was employed to observe general morphology. Additionally, these formalin-fixed, paraffin-embedded slices were subjected to immunohistochemical staining (IHC staining). The slices underwent deparaffinization, followed by a 10-minute treatment with a Peroxidase-Blocking Solution (PBS; Dako, Glostrup, Denmark) to eliminate endogenous peroxidase activity. The slices were rinsed in PBS, followed by a 30-minute incubation at room temperature with primary antibodies against SAMD5 (HPA067811; Sigma-Aldrich, Darmstadt, Germany) and PLK1 (ab189139; Abcam, Cambridge, UK). Subsequently, after PBS washing, the DAKO EnVision + Dual Link System-HRP (DAB+) Kit (DAKO) was employed to detect the antigen-antibody complexes.

Reverse transcription-quantitative polymerase chain reaction (RT-qPCR)

The TRIzol reagent (Invitrogen, San Diego, CA, USA) was employed according to the manufacturer’s instructions to isolate RNA from tissue samples or cultured cells. Then, the PrimeScript RT Reagent Kit (#RR037A; Takara Bio, Otsu, Japan) was utilized to convert the extracted RNA into complementary DNA (cDNA). Primer Premier 5.0 software (Premier Biosoft, Palo Alto, CA, USA) was applied to design primers for the target genes based on their sequences from the NCBI GenBank. SYBR Green Premix Ex Taq Reagent (TaKaRa Bio Inc., Shiga, Japan) on a thermocycler (Analytik-Jena, Jena, Germany) was used to conduct quantitative real-time PCR. With normalization to human glyceraldehyde 3-phosphate dehydrogenase (GAPDH), the relative quantities of RNA were determined using the 2−ΔΔCt method, as described in established protocols.

Immunoblotting

After being washed with cold PBS, tissue samples and cell samples were lysed in cell lysis buffer (Cell Signaling Technologies, Danvers, MA, USA) containing a protease inhibitor cocktail (Beyotime, Shanghai, China) with the help of a tissue homogenizer (Beyotime). Quick Start Bradford reagent (Bio-Rad, Hercules, CA, USA) was employed to determine the total protein content. Following electrophoresis on 10-15% sodium dodecyl sulfate-polyacrylamide gel electrophoresis (SDS-PAGE) gels, 50 µg of separated proteins were electrotransferred from the gel onto nitrocellulose filter membranes (Millipore, Billerica, MA, USA). The membranes were blocked with 5% non-fat milk (in Tris Buffered Saline with Tween 20 (TBST)) for two hours at room temperature. Next, after incubation overnight at 4℃ with primary antibodies, an HRP-labeled IgG antibody (Proteintech, Wuhan, China) was used to incubate with the membrane for one hour at room temperature. The BeyoECL Plus kit (Beyotime) was used to visualize bands. The chemiluminescence signals were captured by an automatic imaging system (Beyotime). The primary antibodies used were as follows: anti-SAMD5 (STJ194894; St John’s Laboratory, London, UK), anti-Ki67 (28074-1-AP; Proteintech), anti-Matrix Metallopeptidase 2 (MMP2) (10373-2-AP; Proteintech), anti-Matrix Metallopeptidase 9 (MMP9) (82854-1-RR; Proteintech), anti-PLK1 (ab189139; Abcam), anti-β-Catenin (51067-2-AP; Proteintech), anti-Cyclin-Dependent Kinase 4 (CDK4) (11026-1-AP; Proteintech), anti-Cyclin-Dependent Kinase 6 (CDK6) (14052-1-AP; Proteintech), anti-Cyclin D1 (26939-1-AP; Proteintech), anti-Cyclin E (11935-1-AP; Proteintech), and anti-GAPDH (60004-1-Ig; Proteintech). The dilutions were 1:1000 to 1:2000.

Cell lines and cell cultivation

Human breast cancer cell lines MDA-MB-231, HCC1937, and BT-483, along with the normal breast epithelial cell lines MCF-10A and MCF-12A, were purchased from the American Type Culture Collection (ATCC, USA). Cell lines were cultivated in the appropriate media containing 10% fetal bovine serum (FBS, Gibco; Thermo Fisher Scientific, Waltham, MA, USA) at 37°C in a humidified incubator supplemented with 5% CO2. Specifically, MCF-10A and MCF-12A were cultivated in DMEM/F12 media containing 5% horse serum, 20 ng/mL epidermal growth factor (EGF), 0.5 µg/mL hydrocortisone, 100 ng/mL cholera toxin, and 10 µg/mL insulin. The MDA-MB-231 cell line was cultivated in Dulbecco's Modified Eagle Medium (DMEM) media containing 10% FBS. The HCC1937 cell line was cultivated in Roswell Park Memorial Institute 1640 medium (RPMI-1640) media containing 10% FBS. The BT-483 cell line was cultivated in RPMI-1640 media containing 20% FBS. Cells were cultured at 37°C in an incubator supplemented with 5% CO2 under 95% humidity saturation.

Cell transfection

SAMD5 overexpression or PLK1 plasmids were introduced into breast cancer cell lines to achieve SAMD5 or PLK1 overexpression, while PLK1 knockdown was performed using three specific small interfering RNAs (si-PLK1-1, si-PLK1-2, si-PLK1-3). A non-targeting scramble siRNA (si-NC) served as the negative control. Additionally, PLK1 overexpression was carried out in separate experiments. The transfections were conducted using OriTrans®PEI-DNA (for plasmid transfection) and OriTrans®PEI-X (for siRNA transfection) (ORI2305, ORI2311; Ori-Bio, Changsha, China), according to the manufacturer’s protocol. After transfection for 48 hours, cells were harvested for further experiments.

Cell Counting Kit-8 (CCK-8) assay

Using the CCK-8 assay, cell viability was evaluated. Transfected MDA-MB-231 and HCC1937 cell lines were initially plated onto 96-well plates (2 × 10⁴ cells/well). Following seeding, cells underwent the specified transfection or treatment procedures. Subsequently, each well was supplemented with 10 μL of CCK-8 reagent (Beyotime), followed by a four-hour incubation. A microplate reader was applied to measure the absorbance at a wavelength of 450 nm.

Colony formation

To conduct the colony formation assay, logarithmic growth phase cells were used for transfection and/or treatment, and then seeded into six-well plates (1.5 × 10³ cells/well). Colony formation was carried out by culturing cells for 18 days. Subsequently, after 10 minutes of fixing with methanol, cells were subjected to staining with 0.2% crystal violet before photographing and counting the cell colonies. Each assay was independently performed in triplicate, with every experiment conducted in triplicate.

Transwell assay detecting invasion

For invasion assays, Matrigel-coated (BD Biosciences, Franklin Lakes, NJ, USA) Transwell chambers (Corning Inc., Corning, NY, USA) were utilized. After collection, transfected cells were re-suspended in serum-free RPMI-1640 media. Then, the top chamber of a 24-well Transwell insert was supplemented with a total of 100,000 cells, with the bottom chamber containing RPMI-1640 media, added with 20% FBS, serving as a chemoattractant. After a 48-hour incubation, cotton swabs were employed to eliminate cells that had not invaded from the top surface of the membrane. The cells migrating to the underside were subjected to fixing with 100% methanol, staining with 0.2% crystal violet, and visualized under a microscope.

Xenograft tumor model in nude mice

Animal studies were performed following the Animal Research Reporting of In Vivo Experiments (ARRIVE) guidelines and were approved by the Ethics Committee of the Sichuan Academy of Medical Sciences and Sichuan Provincial People’s Hospital (approval no. TYL20210817). To investigate the in vivo effects of SAMD5 and PLK1 overexpression, a subcutaneous tumor model was constructed in nude mice. Four-to-six-week-old BALB/c nude mice (weighing 20-24 g) were purchased from SLAC laboratory animal company (Changsha, China) and kept in specific pathogen-free conditions. Each mouse was subcutaneously injected with breast cancer cells (5 × 10^6 ^cells) suspended in 0.1 mL of culture media. One week post-injection, the tumor sites were injected with lentiviruses (1 × 10^6^ TU in 20 μL) once per week to overexpress SAMD5 and PLK1. The mice were allocated into three groups (each with six mice): Group 1 (Lv-Vector), Group 2 (Lv-SAMD5), and Group 3 (Lv-SAMD5 + Lv-PLK1). The lentivirus was obtained as previously described [22]. Over the course of four weeks, tumor growth was monitored. After four weeks, tumors were excised and measured. Tumor volume and size were documented to assess the effects of gene overexpression on tumor progression. Protein from tumor tissues was isolated to determine proliferation-associated protein expression.

Statistical analysis

Experimental data were represented as means ± standard deviation (SD). Differences among groups were identified using one-way analysis of variance (ANOVA), followed by Tukey’s post hoc test. The GraphPad Prism 8.0 software (GraphPad Software, San Diego, CA, USA) was utilized for all statistical analyses. A p-value <0.05 was considered statistically significant.

## Results

Identifying key genes affecting breast cancer by differential expression analysis

**Figure 1:**
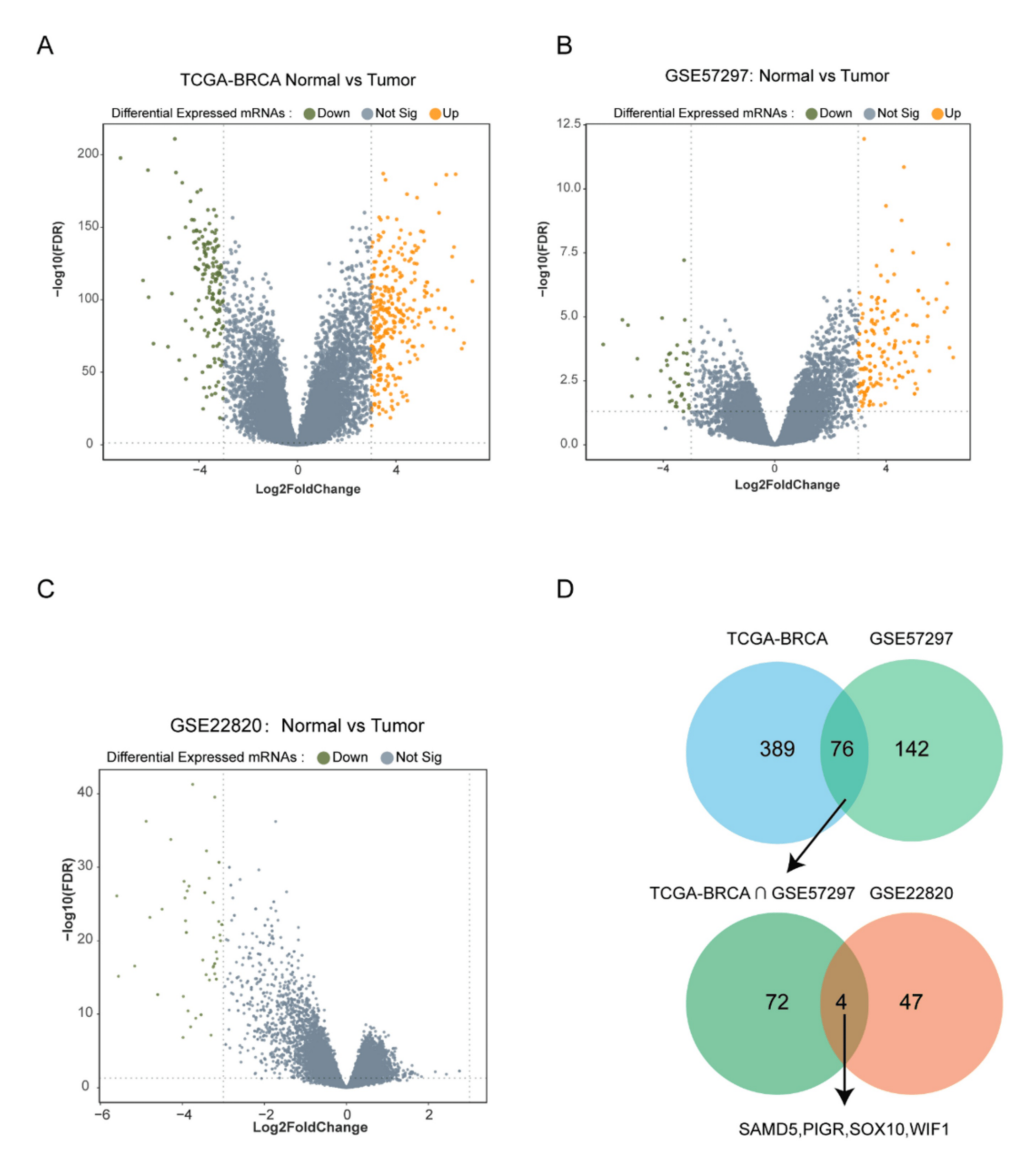
Differentially expressed genes between breast cancer and normal samples (A) Microarray data were obtained from Xena (https://xenabrowser.net/datapages/) using the IlluminaHiSeq platform, which included 114 normal tissue samples and 1,104 breast cancer samples. Differential gene expression was analyzed using The Cancer Genome Atlas Breast Invasive Carcinoma (TCGA-BRCA) dataset with the Linear Models for Microarray and RNA-Seq Data (limma) package. (B)-(C) Differential gene expression was further analyzed in the Gene Expression Omnibus (GEO) datasets GSE57297 and GSE22820 using the limma package. (D) The Venn diagram shows the four common differentially expressed genes (DEGs) identified in the TCGA-BRCA, GSE57297, and GSE22820 datasets. SAMD5: Sterile Alpha Motif Domain-Containing 5; PIGR: Polymeric Immunoglobulin Receptor; SOX10: SRY-Box Transcription Factor 10; WIF1: WNT Inhibitory Factor 1; mRNA: messenger RNA

To identify key factors influencing breast cancer, differential expression analysis was conducted using multiple datasets. Initially, TCGA-BRCA data from Xena (https://xenabrowser.net/datapages/), which contains 114 tumor-free breast tissue samples and 1,104 breast cancer samples, was analyzed. Differential gene expression was determined using the TCGA-BRCA dataset analyzed with the limma package, revealing 154 up-regulated genes (log_2_ Fold Change (log_2_FC) > 3, adjusted p < 0.05) and 311 down-regulated genes (log_2_FC < -3, adjusted p < 0.05) (Figure 1A). Further analysis was conducted using the GSE57297 dataset, obtaining 41 up-regulated genes (log_2_FC > 3, adjusted p < 0.05) and 177 down-regulated genes (log_2_FC < -3, adjusted p < 0.05) (Figure 1B). Additionally, the GSE22820 dataset, which includes 10 non-cancerous specimens and 176 breast cancer specimens, was used, identifying zero up-regulated genes (log_2_FC > 3, adjusted p < 0.05) and 51 down-regulated genes (log_2_FC < -3, adjusted p < 0.05) (Figure 1C). Comparing these with the previously identified genes from the TCGA-BRCA and GSE57297 datasets revealed four distinct down-regulated genes: SAMD5, Polymeric Immunoglobulin Receptor (PIGR), SRY-Box Transcription Factor 10 (SOX10), and WNT Inhibitory Factor 1 (WIF1) (Figure 1D).

**Figure 2:**
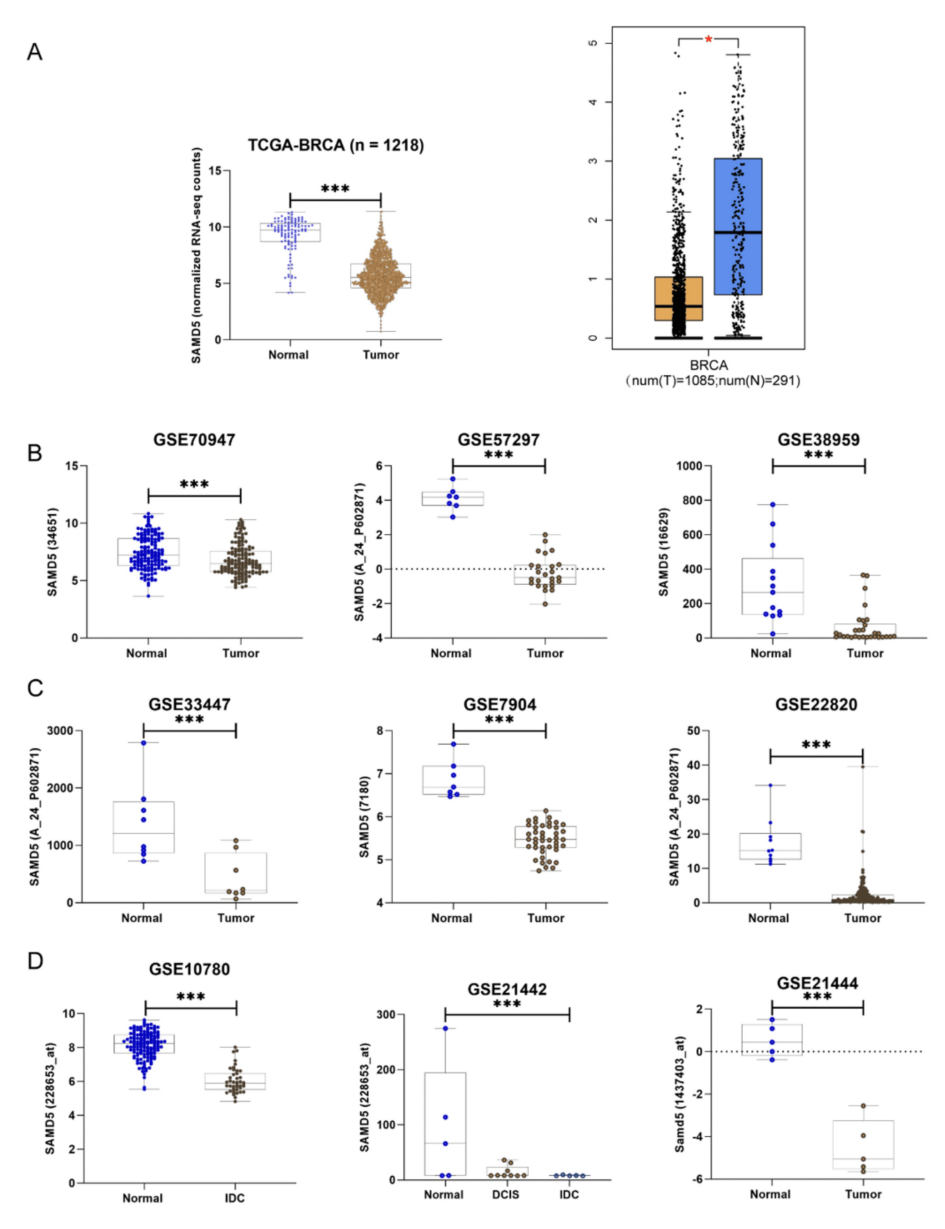
Screening and validation of Sterile Alpha Motif Domain-Containing 5 (SAMD5) gene expression in breast cancer (A) The expression of Sterile Alpha Motif Domain Containing 5 (SAMD5) was analyzed in The Cancer Genome Atlas Breast Invasive Carcinoma (TCGA-BRCA) breast cancer dataset (normal = 114, tumor = 1,104) and validated using Gene Expression Profiling Interactive Analysis (GEPIA2) with integrated TCGA-BRCA/Genotype-Tissue Expression (GTEx) data (normal = 291, tumor = 1,085). The expression of SAMD5 was examined across multiple Gene Expression Omnibus (GEO) datasets, including GSE70947, GSE57297, and GSE38959 (B); GSE33447, GSE7904, and GSE22820 (C); and GSE10780, GSE21442, and GSE21444 (D). ***p < 0.001

The expression level of SAMD5, which belongs to the SAMD family involved in cell differentiation and potentially regulating cell proliferation and apoptosis [20], was analyzed. Using the TCGA-BRCA dataset (normal = 114, tumor = 1104), significant downregulation of SAMD5 was observed (log_2_FC = -3.53, p = 7.057 × 10^-92^) and validated with integrated TCGA-BRCA/Genotype-Tissue Expression (GTEx) data (normal = 291, tumor = 1085) via Gene Expression Profiling Interactive Analysis (GEPIA2), confirming SAMD5's significantly lower expression in tumor tissues (Figure 2A). Further validation of SAMD5 expression was performed across multiple Gene Expression Omnibus (GEO) datasets. Significant downregulation of SAMD5 was observed in GSE70947 (log_2_FC = -2.39, p = 3.98 × 10^-5^), GSE57297 (log_2_FC = -4.47, p = 4.03 × 10^-14^), GSE38959 (log_2_FC = -3.36, p = 1.58 × 10^-6^), GSE33447 (log_2_FC = -2.12, p = 6.59 × 10^-4^), GSE7904 (log_2_FC = -1.40, p = 5.32 × 10^-12^), and GSE22820 (log_2_FC = -3.99, p = 4.43 × 10^-13^) (Figures 2B-2C). Additional datasets, such as GSE10780, revealed significant downregulation of SAMD5 in invasive ductal carcinoma (IDC) (log_2_FC = -2.093, p = 5.57 × 10^-35^) compared to normal breast tissue. Similarly, GSE21442 showed downregulation in both non-IDC in situ and IDC (log_2_FC = -1.81, p = 4.70 × 10^-2^). The GSE21444 dataset, which includes an animal model of breast cancer induced by oncogenic transcription factors, supported the significant downregulation of SAMD5 (log_2_FC = -5.055, p = 2.80 × 10^-5^) (Figure 2D).

**Figure 3:**
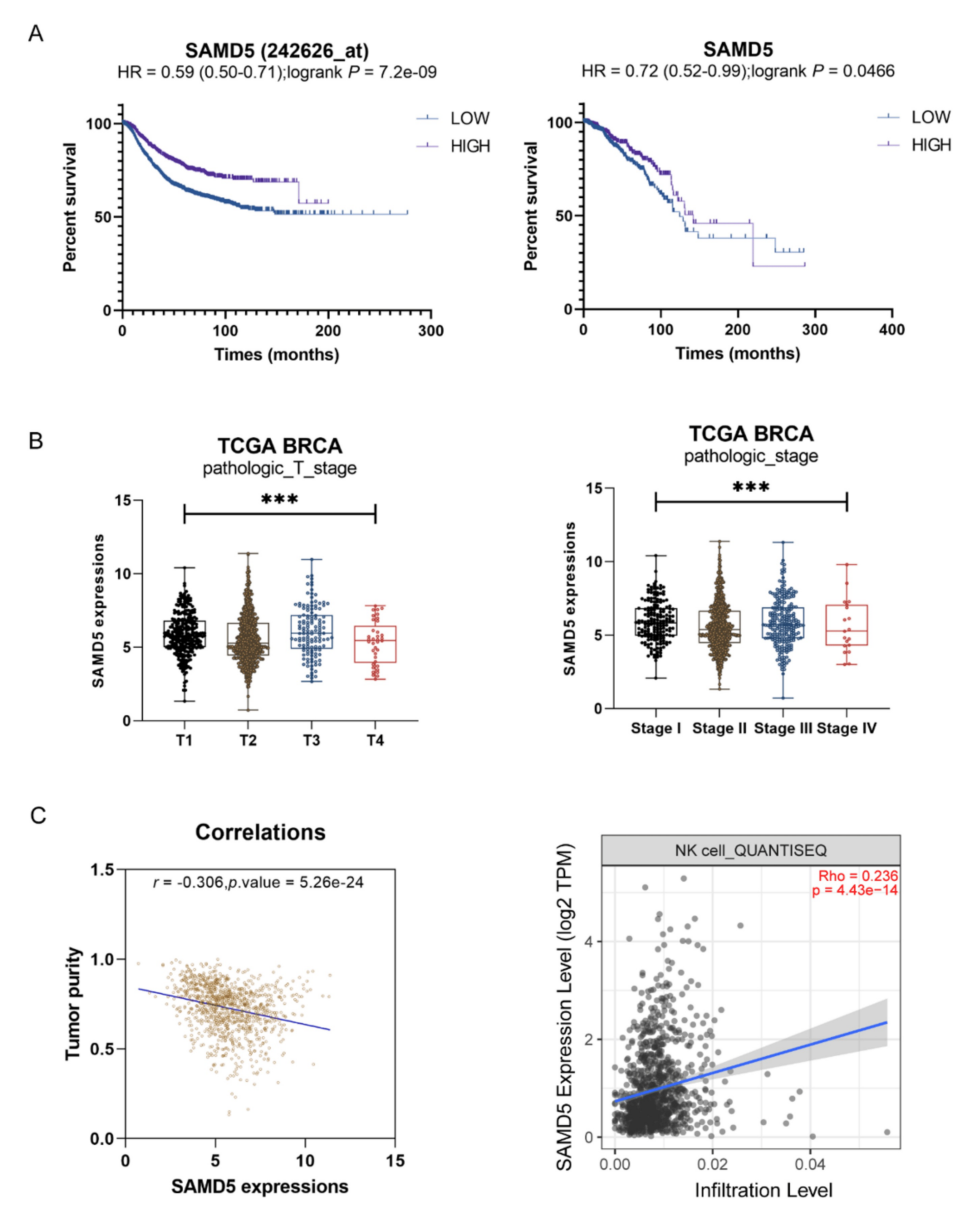
Clinical analysis of Sterile Alpha Motif Domain-Containing 5 (SAMD5) in breast cancer (A) The prognostic value of Sterile Alpha Motif Domain-Containing 5 (SAMD5) expression was evaluated using the Kaplan-Meier Plotter (KMplot) online tool, which analyzed both gene chip data and RNA sequencing (RNAseq) data to determine its impact on overall survival (OS) in breast cancer patients. (B) The relationship between SAMD5 expression and clinical features was examined using data from The Cancer Genome Atlas Breast Invasive Carcinoma (TCGA-BRCA) dataset, including the analysis of its expression across different pathological stages and Tumor-Node-Metastasis (TNM) classifications. (C) The association of SAMD5 expression with tumor purity and immune cell infiltration was investigated using the Tumor Immune Estimation Resource (TIMER) (http://timer.comp-genomics.org/), which analyzed the correlation between SAMD5 expression and the infiltration of cytotoxic natural killer (NK) cells. ***p < 0.001

The prognostic value of SAMD5 was evaluated using the KMplot online tool, which analyzed both gene chip and RNAseq data to determine its association with overall survival in patients with breast cancer. High SAMD5 expression was found to be a protective factor for overall survival (hazard ratio < 1, p < 0.05) (Figure 3A). The data from the TCGA-BRCA dataset were employed to examine the correlation between SAMD5 expression levels and clinical features. SAMD5 expression was found to significantly decrease with pathological stage progression (p = 7.180 × 10^-3^) and TNM classification, with the lowest expression at T4 (p = 5.669 × 10^-5^) (Figure 3B). Furthermore, the correlation of SAMD5 expression levels with tumor purity and infiltration of immune cells was investigated. SAMD5 expression levels were found to exhibit a significant negative correlation with tumor purity (r = -0.306, p = 5.261 × 10^-24^). Based on Tumor Immune Estimation Resource (TIMER) analysis, SAMD5 expression levels were shown to be positively correlated with cytotoxic natural killer (NK) cell infiltration (Figure 3C).

**Figure 4:**
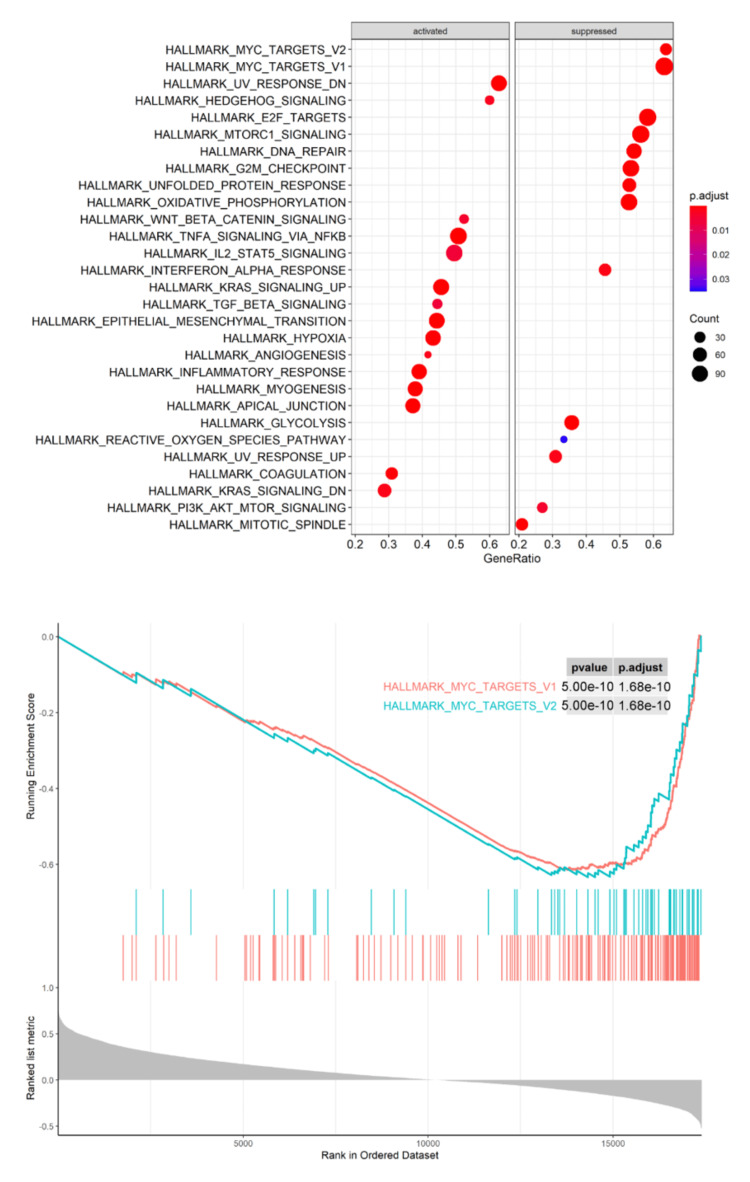
Kyoto Encyclopedia of Genes and Genomes (KEGG) analysis of Sterile Alpha Motif Domain-Containing 5 (SAMD5) expression related genes in The Cancer Genome Atlas Breast Invasive Carcinoma (TCGA-BRCA) Pearson's correlation analysis between Sterile Alpha Motif Domain-Containing 5 (SAMD5) expression and other genes was performed using data from The Cancer Genome Atlas Breast Invasive Carcinoma (TCGA-BRCA) dataset (n = 1,217), covering 20,530 gene expressions. The correlated genes were subsequently subjected to Kyoto Encyclopedia of Genes and Genomes (KEGG) enrichment analysis.

The TCGA-BRCA dataset was applied to perform Pearson's correlation analysis between SAMD5 expression and 20,530 gene expressions. The related genes were subjected to Kyoto Encyclopedia of Genes and Genomes (KEGG) enrichment analysis (Figure 4), revealing that SAMD5 genes were significantly negatively correlated and enriched with cell cycle proliferation pathways, particularly within the HALLMARK_MYC_TARGETS_V1/V2 pathways. These findings suggest a potential link between SAMD5 and the Myc signaling pathway, indicating that SAMD5 might play a role in regulating this critical pathway within breast carcinoma.

SAMD5 expression within breast cancer clinical tissue samples and cell lines

**Figure 5:**
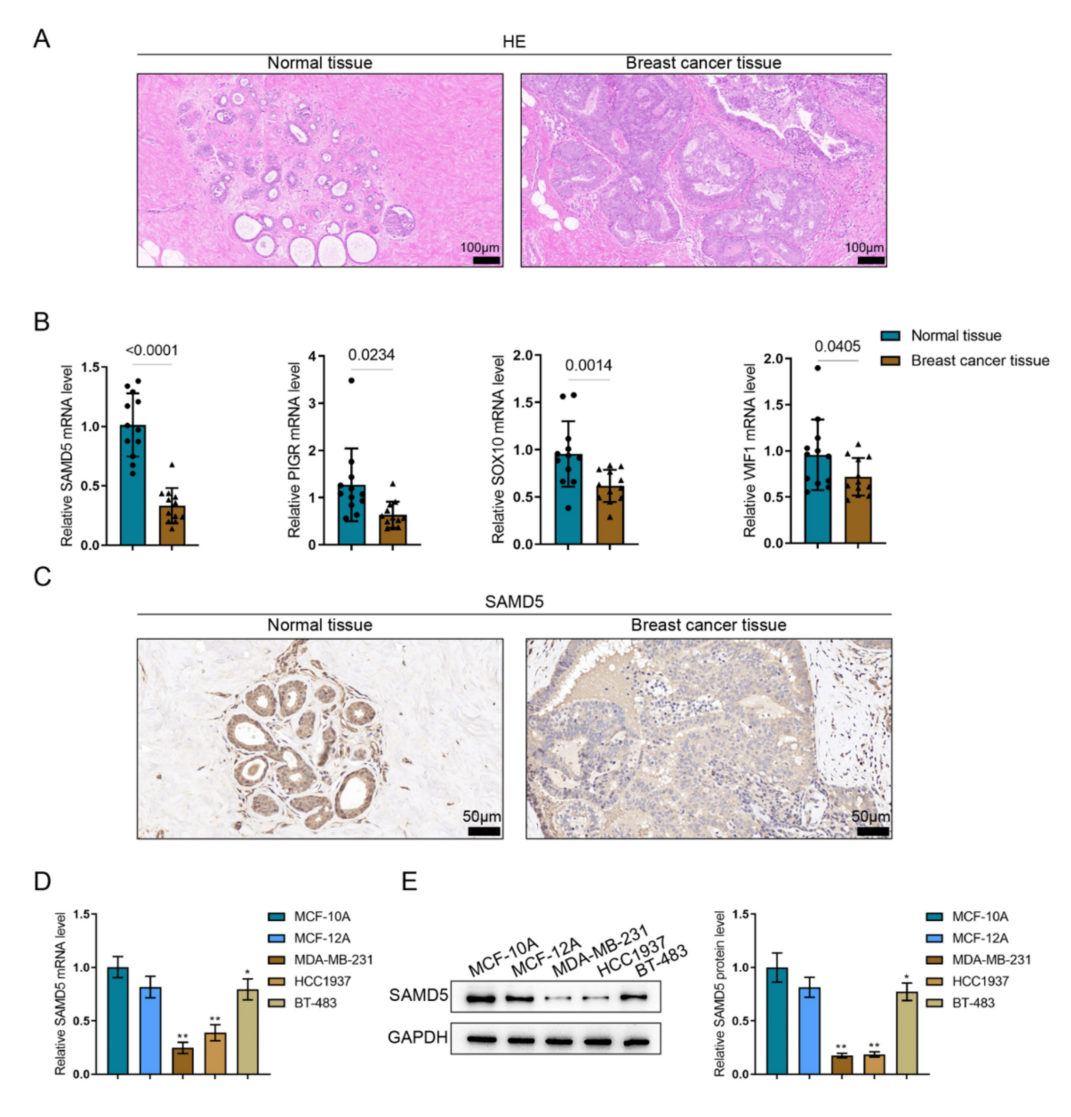
Expression of Sterile Alpha Motif Domain-Containing 5 (SAMD5) in breast cancer clinical tissues and cell lines (A) Breast cancer tissues and adjacent normal breast tissues were collected. Hematoxylin and eosin (H&E) staining was performed to confirm pathological status. (B) The messenger RNA (mRNA) expression levels of Sterile Alpha Motif Domain-Containing 5 (SAMD5), Polymeric Immunoglobulin Receptor (PIGR), SRY-Box Transcription Factor 10 (SOX10), and Wnt Inhibitory Factor 1 (WIF1) were determined in tissue samples using Reverse Transcription Quantitative Polymerase Chain Reaction (RT-qPCR) (n = 12). (C) The protein abundance of SAMD5 in tissues was assessed using immunohistochemistry (IHC). (D) The mRNA levels of SAMD5 in normal breast epithelial cells (MCF-10A and MCF-12A) and breast cancer cell lines (MDA-MB-231, HCC1937, and BT-483) were determined using RT-qPCR. (E) The protein expression levels of SAMD5 were examined in the cell lines using immunoblotting. *p < 0.05; **p < 0.01

Next, breast cancer tissues and adjacent normal tissues were collected and validated using H&E staining (Figure 5A). The mRNA expression levels of overlapping downregulated genes - SAMD5, PIGR, SOX10, and WIF1 - were determined in tissue samples using RT-qPCR (n = 12). Figure 5B shows that the four genes were significantly downregulated in breast cancer samples, with SAMD5 being the most downregulated. Therefore, SAMD5 was selected for the following investigations. The protein abundance of SAMD5 in clinical breast cancer and paired non-tumor tissues was assessed using immunohistochemistry (IHC); as shown in Figure 5C, SAMD5 levels were remarkably lower in breast carcinoma samples. Next, SAMD5 mRNA expression was determined in non-cancerous breast epithelial cells (MCF-10A and MCF-12A) and breast carcinoma cell lines (MDA-MB-231, HCC1937, and BT-483); Figure 5D shows that compared with MCF-10A and MCF-12A, SAMD5 mRNA expression was significantly downregulated in MDA-MB-231, HCC1937, and BT-483 cell lines. Similarly, SAMD5 protein levels were decreased in MDA-MB-231, HCC1937, and BT-483 cells relative to MCF-10A and MCF-12A cells (Figure 5E).

In vitro effects of SAMD5 upon breast cancer cell phenotypes

**Figure 6:**
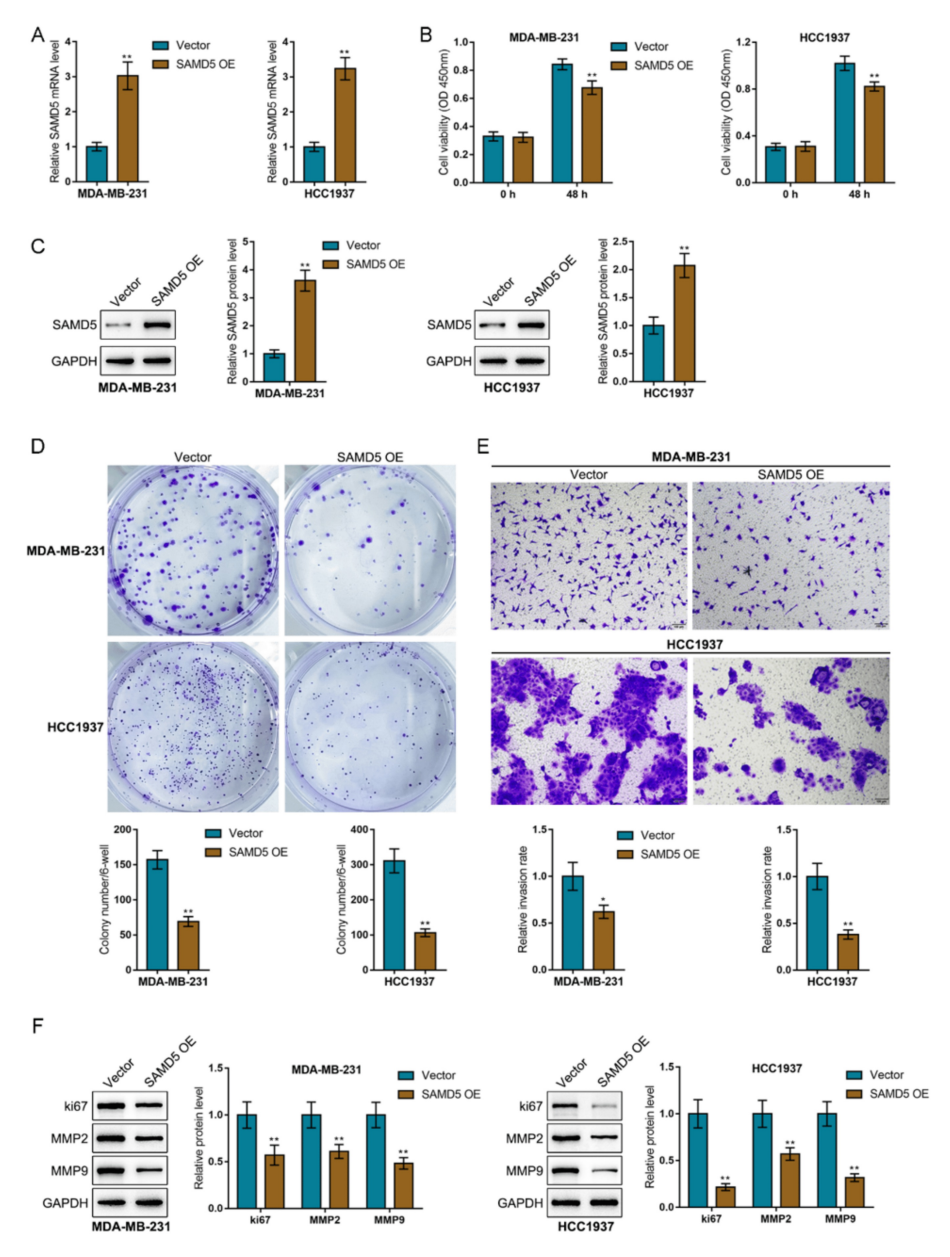
In vitro effects of Sterile Alpha Motif Domain-Containing 5 (SAMD5) on breast cancer cell phenotypes (A) and (C) Sterile Alpha Motif Domain-Containing 5 (SAMD5) overexpression was achieved in two triple-negative breast cancer cell lines (MDA-MB-231 and HCC1937) by introducing SAMD5 overexpression (OE), which was validated using Reverse Transcription Quantitative Polymerase Chain Reaction (RT-qPCR) and immunoblotting. (B) MDA-MB-231 and HCC1937 cells were transfected with SAMD5 OE and examined for cell viability using the Cell Counting Kit-8 (CCK-8) assay. (D) Colony formation was assessed in the transfected cells. (E) Cell invasion was evaluated using the Transwell assay. (F) The protein levels of the proliferation marker Ki67 and the invasion markers Matrix Metalloproteinase 2 (MMP2) and Matrix Metalloproteinase 9 (MMP9) were determined using immunoblotting. *p < 0.05; **p < 0.01

After confirming the downregulation of SAMD5 in breast cancer, SAMD5 overexpression was achieved in two TNBC cell lines (MDA-MB-231 and HCC1937) by introducing SAMD5 overexpression and validated using RT-qPCR and immunoblotting for further functional investigations (Figure 6A and Figure 6C). Then, MDA-MB-231 and HCC1937 cells were transfected with SAMD5 overexpression and examined for cell phenotypes. SAMD5 overexpression considerably suppressed cell viability (Figure 6B), colony formation (Figure 6D), and invasive capability (Figure 6E) in both cell lines. Consistently, SAMD5 overexpression markedly reduced Ki67, MMP2, and MMP9 protein levels in breast cancer cells relative to the control group (Figure 6F).

PLK1 is negatively correlated with SAMD5

**Figure 7:**
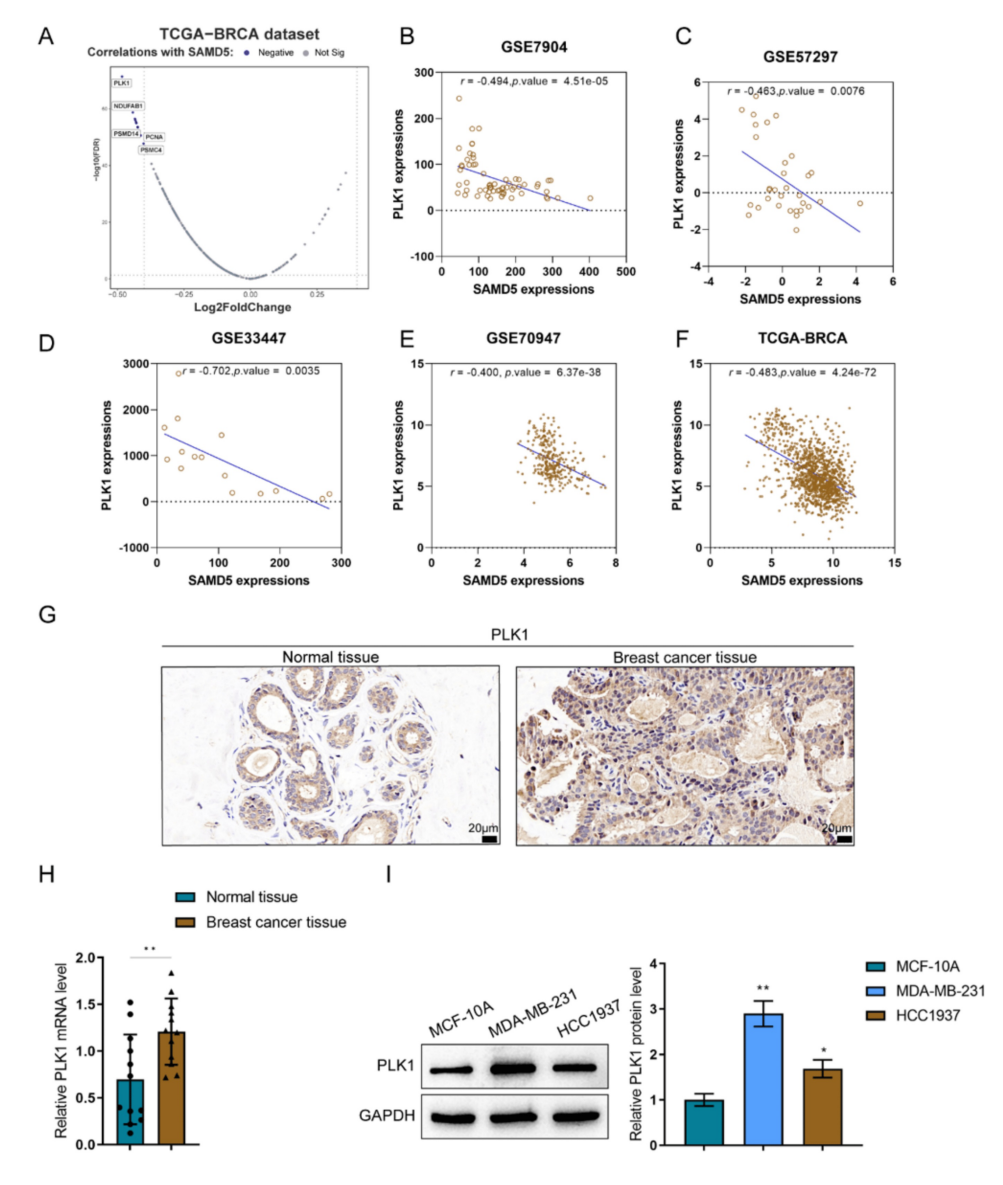
Polo-like Kinase 1 (PLK1) is negatively correlated with Sterile Alpha Motif Domain-Containing 5 (SAMD5) (A) The 258 genes of the Hallmark MYC Targets Version 1/Version 2 (HALLMARK_MYC_TARGETS_V1/V2) pathway were analyzed for expression correlation with Sterile Alpha Motif Domain-Containing 5 (SAMD5). (B)-(F) Correlation analysis of Polo-like Kinase 1 (PLK1) and SAMD5 was conducted using data from the Gene Expression Omnibus (GEO) datasets GSE70947, GSE57297, GSE33447, GSE7904, and The Cancer Genome Atlas Breast Invasive Carcinoma (TCGA-BRCA). (G)-(H) Immunohistochemistry (IHC) staining and Reverse Transcription Quantitative Polymerase Chain Reaction (RT-qPCR), PLK1 expression was higher in breast cancer tissues compared to adjacent non-tumor tissues. *p < 0.05; **p < 0.01

Regarding the mechanism underlying SAMD5 function in breast cancer, the 258 genes of the HALLMARK_MYC_TARGETS_V1/V2 pathway were analyzed for factors correlated with SAMD5; 13 genes were found to be remarkably negatively correlated with SAMD5 (r < -0.40, p < 0.05), among which PLK1 had the strongest negative correlation (Figure 7A). According to GSE7904, GSE57297, GSE33447, GSE70947, and TCGA-BRCA, PLK1 and SAMD5 were negatively correlated (Figures 7B-7F). PLK1 exhibits overexpression in breast cancer, particularly in TNBC [23]; therefore, PLK1 was selected for further investigations. As shown by IHC staining and RT-qPCR, PLK1 expression was higher in breast cancer tissues compared to adjacent non-tumor tissues (Figures 7G-7H). Moreover, the expression of PLK1 was also upregulated in breast cancer cell lines compared to normal breast epithelial cells (Figure 7I).

In vitro effects of PLK1 knockdown upon breast cancer cell phenotypes

**Figure 8:**
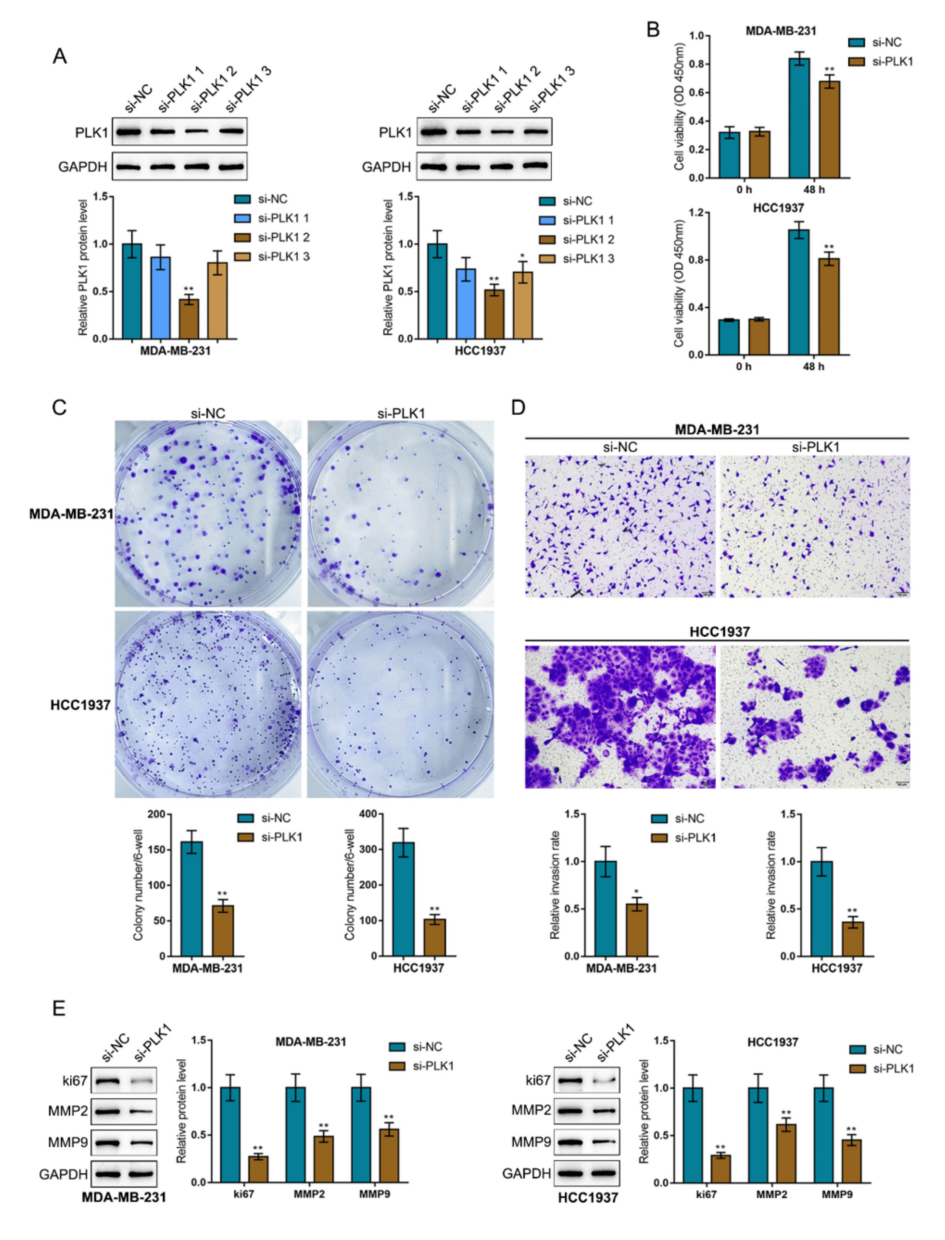
In vitro effects of Polo-like Kinase 1 (PLK1) knockdown on breast cancer cell phenotypes (A) Polo-like Kinase 1 (PLK1) knockdown was achieved in two triple-negative breast cancer cell lines (MDA-MB-231 and HCC1937) by introducing small interfering RNA against PLK1 (siRNA-PLK1-1/2/3), which was validated using immunoblotting. si-PLK1-2 was selected for further investigations due to its higher efficiency. (B) MDA-MB-231 and HCC1937 cells were transfected with si-PLK1 and examined for cell viability using the Cell Counting Kit-8 (CCK-8) assay. (C) Colony formation was assessed in the transfected cells. (D) Cell invasion was evaluated using the Transwell assay. (E) The protein levels of proliferation marker Ki67 and invasion markers Matrix Metalloproteinase 2 (MMP2) and Matrix Metalloproteinase 9 (MMP9) were determined using immunoblotting. *p < 0.05; **p < 0.01

Given the upregulation of PLK1 in breast cancer, PLK1 knockdown was achieved in two TNBC cell lines (MDA-MB-231 and HCC1937) by introducing small interfering RNA against PLK1 (si-PLK1-1/2/3) and validated using immunoblotting. si-PLK1-2 was used in subsequent investigations due to better efficiency (Figure 8A). Then, after being transfected with si-PLK1, MDA-MB-231 and HCC1937 cells were examined for cell phenotypes. PLK1 silencing dramatically suppressed cell viability (Figure 8B), colony formation (Figure 8C), and cell invasion (Figure 8D) relative to the si-NC group. Regarding cancer proliferation and invasion-associated markers, Ki67, MMP2, and MMP9 protein levels were significantly decreased following PLK1 knockdown relative to the si-NC group (Figure 8E).

PLK1 overexpression mitigates SAMD5 effects in breast cancer cells

**Figure 9:**
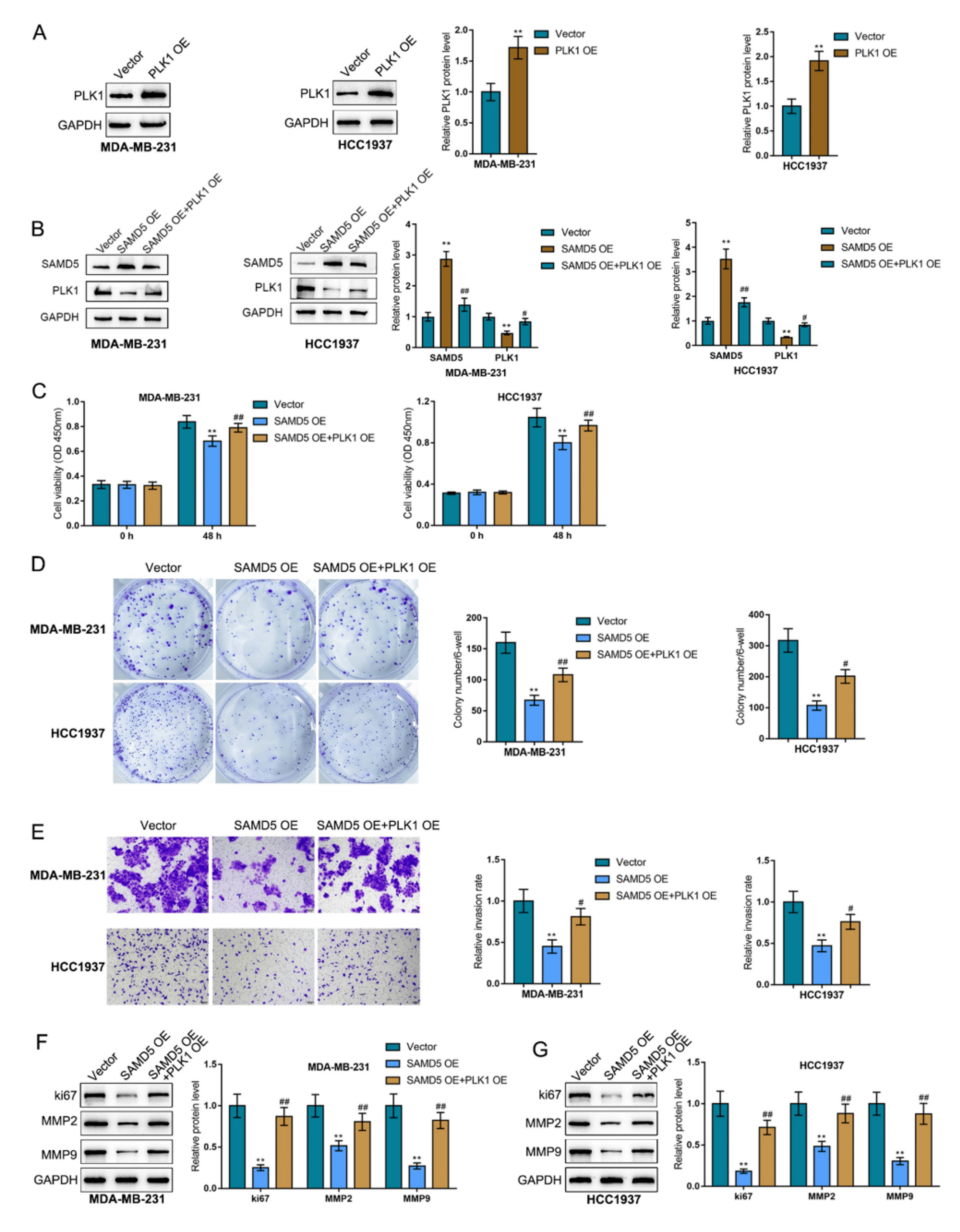
Polo-like Kinase 1 (PLK1) overexpression partially attenuates Sterile Alpha Motif Domain-Containing 5 (SAMD5) overexpression effects on breast cancer cells (A) Polo-like Kinase 1 (PLK1) overexpression was achieved in two triple-negative breast cancer cell lines (MDA-MB-231 and HCC1937) by introducing PLK1 overexpression (OE), which was validated using immunoblotting. (B) MDA-MB-231 and HCC1937 cells were then co-transfected with Sterile Alpha Motif Domain-Containing 5 (SAMD5) overexpression (OE) and PLK1 OE, and the protein levels of SAMD5 were analyzed using immunoblotting. (C) Cell viability was assessed using the Cell Counting Kit-8 (CCK-8) assay. (D) Colony formation was evaluated in the transfected cells. (E) Cell invasion was measured using the Transwell assay. (F)-(G) The protein levels of proliferation marker Ki67 and invasion markers Matrix Metalloproteinase 2 (MMP2) and Matrix Metalloproteinase 9 (MMP9) were determined using immunoblotting. **p < 0.01 vs. vector group; #p < 0.05; ##p < 0.01 vs. SAMD5 group

Since SAMD5 negatively correlates with PLK1, the dynamic effects of SAMD5 and PLK1 on breast cancer cells were explored to investigate whether PLK1 mediates SAMD5 functions in breast cancer. PLK1 overexpression was achieved in two TNBC cell lines (MDA-MB-231 and HCC1937) by introducing PLK1 overexpression and validated using immunoblotting (Figure 9A). Then, after co-transfection with SAMD5 overexpression and PLK1 overexpression, MDA-MB-231 and HCC1937 cell lines were analyzed for SAMD5 protein levels. Figure 9B shows that SAMD5 overexpression increased SAMD5 but decreased PLK1, whereas PLK1 overexpression decreased SAMD5 but increased PLK1; the effects of SAMD5 overexpression on SAMD5 and PLK1 could be partially reversed by PLK1 overexpression. Regarding cell phenotypes, PLK1 overexpression facilitated cell viability (Figure 9C), colony formation (Figure 9D), and invasive capability (Figure 9E); additionally, PLK1 overexpression partially attenuated the effects of SAMD5 overexpression on cell viability, colony formation, and cell invasion (Figures 9C-9E) in breast cancer cells. Regarding cancer biomarkers, Ki67, MMP2, and MMP9 protein levels were decreased by SAMD5 but increased by PLK1; PLK1 overexpression significantly alleviated the inhibitory effects of SAMD5 on these factors (Figures 9F-9G). Therefore, PLK1 may mediate the functions of SAMD5 in breast cancer cells.

SAMD5 interacts with PLK1 to modulate the c-Myc signaling in breast cancer cells

**Figure 10:**
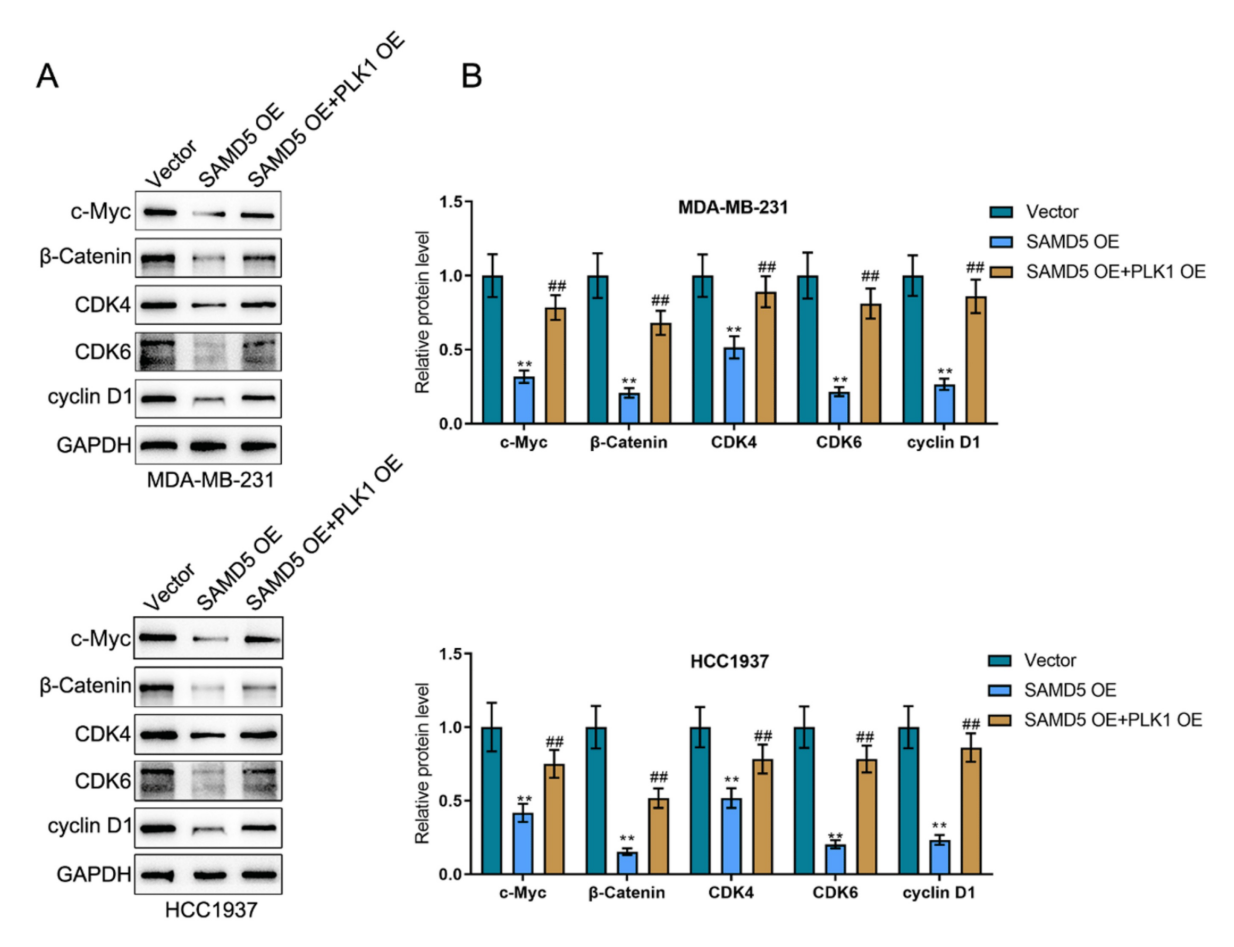
Sterile Alpha Motif Domain-Containing 5 (SAMD5) interacts with Polo-like Kinase 1 (PLK1) to modulate the c-Myc signaling in breast cancer cells (A)-(B) MDA-MB-231 and HCC1937 cells were co-transfected with Sterile Alpha Motif Domain-Containing 5 (SAMD5) overexpression (OE) and Polo-like Kinase 1 (PLK1) OE and examined for the protein levels of MYC Proto-Oncogene (c-Myc), β-catenin, Cyclin-Dependent Kinase 4 (CDK4), Cyclin-Dependent Kinase 6 (CDK6), and Cyclin D1 using immunoblotting. **p < 0.01 vs. vector group; ##p < 0.01 vs. SAMD5 group

Given the correlation of SAMD5 with the HALLMARK_MYC_TARGETS_V1/V2 pathway, the dynamic effects of SAMD5/PLK1 on the c-Myc signaling pathway were investigated. After co-transfection with SAMD5 overexpression and PLK1 overexpression, MDA-MB-231 and HCC1937 cell lines were analyzed for c-Myc, β-catenin, CDK4, CDK6, and Cyclin D1 proteins. Figures 10A-10B show that the protein levels of c-Myc, β-catenin, CDK4, CDK6, and Cyclin D1 were drastically decreased by SAMD5 overexpression but markedly elevated by PLK1 overexpression, suggesting that PLK1 mediates the effects of SAMD5 on the c-Myc signaling pathway.

Dynamic effects of SAMD5 and PLK1 on tumor growth in a mouse model

**Figure 11:**
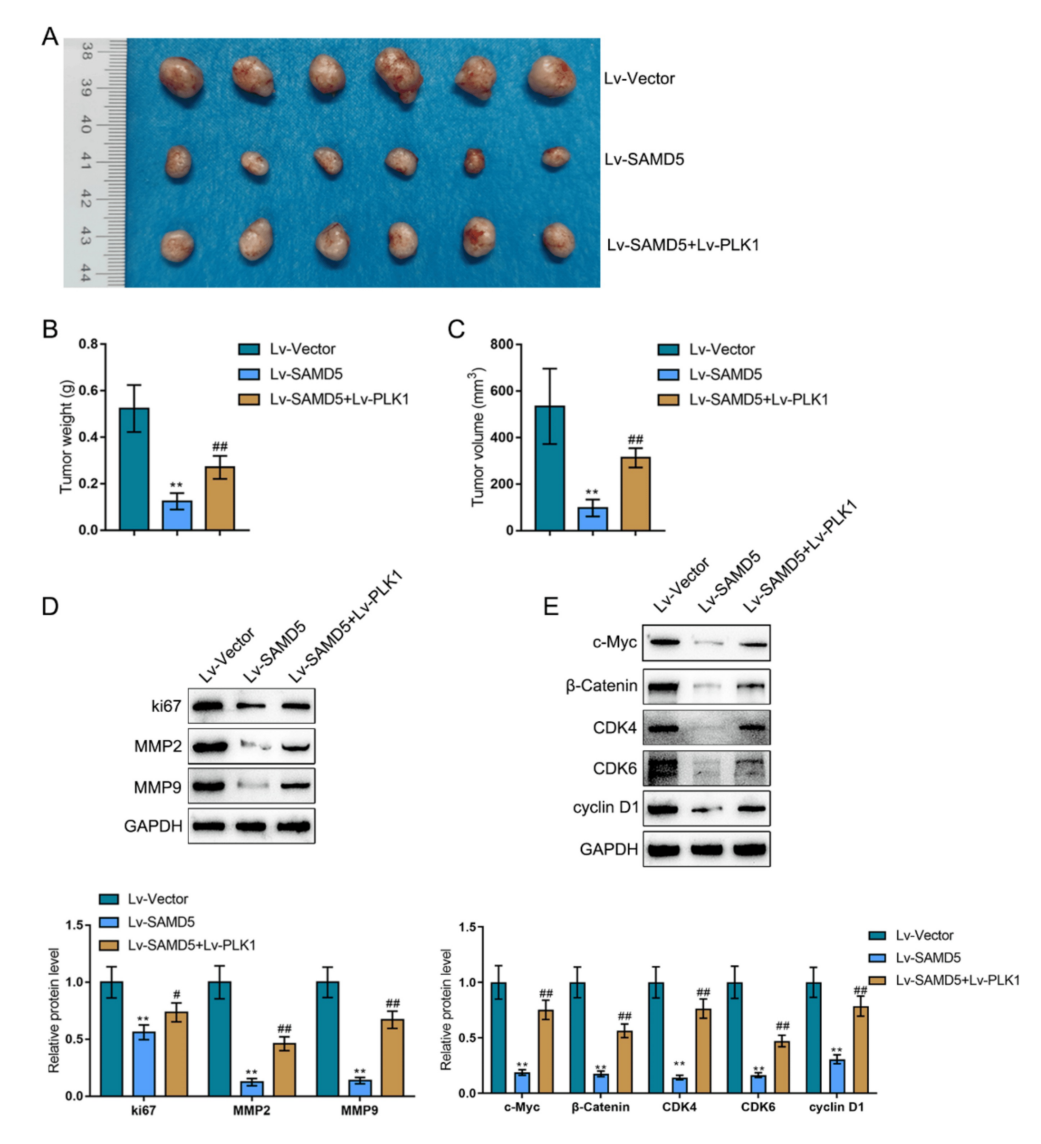
Dynamic effects of Sterile Alpha Motif Domain-Containing 5 (SAMD5) and Polo-like Kinase 1 (PLK1) on tumor growth in a mouse model (A) A xenograft tumor model was established in nude mice, and lentivirus-mediated overexpression of Sterile Alpha Motif Domain-Containing 5 (SAMD5) and/or Polo-like Kinase 1 (PLK1) was introduced into the tumors. (B)-(C) Tumor weight and volume were measured. (D) The protein levels of MYC Proto-Oncogene (c-Myc), β-catenin, Cyclin-Dependent Kinase 4 (CDK4), Cyclin-Dependent Kinase 6 (CDK6), and Cyclin D1 in tumor tissues were determined using immunoblotting. **p < 0.01 vs. vector group; #p < 0.05; ##p < 0.01 vs. SAMD5 group

Lastly, a xenograft tumor model was established in nude mice, where lentivirus-mediated overexpression of SAMD5 and/or PLK1 was introduced into tumors, and the dynamic effects of SAMD5/PLK1 were investigated. SAMD5 overexpression significantly decreased tumor weight and volume, whereas PLK1 overexpression partially attenuated these effects (Figures 11A-11C). Consistently, Ki67, MMP2, MMP9, c-Myc, β-catenin, CDK4, CDK6, and Cyclin D1 protein levels in tumor tissues were significantly decreased by SAMD5 overexpression but partially increased by PLK1 overexpression (Figures 11D-11E).

## Discussion

In this study, SAMD5 was proven to be a significantly downregulated factor in breast carcinoma by integrative differential expression analysis, with a close correlation with the c-Myc signaling. SAMD5 expression levels were shown to be dramatically decreased in breast carcinoma tissue samples and cells compared to adjacent normal tissue samples and non-cancerous breast epithelial cell lines. Functional assays revealed that overexpression of SAMD5 in TNBC cells inhibited cell viability, colony formation, and invasion, and reduced the levels of proliferation and invasion markers, including Ki67, MMP2, and MMP9. Mechanistic studies indicated that SAMD5 negatively correlated with PLK1, a gene overexpressed in breast carcinoma, especially in triple-negative subtypes. Knockdown of PLK1 in breast carcinoma cell lines suppressed cell viability, colony formation, and invasion, while PLK1 overexpression attenuated the inhibitory effects of SAMD5 overexpression. Additionally, SAMD5 overexpression was shown to downregulate key proteins in the c-Myc signaling pathway, including c-Myc, β-catenin, CDK4, CDK6, and Cyclin D1, effects that were counteracted by PLK1 overexpression. In vivo studies using a xenograft tumor model in nude mice demonstrated that SAMD5 overexpression reduced tumor weight and volume, with these effects being partially reversed by PLK1 overexpression, highlighting the interaction between SAMD5 and PLK1 in modulating breast carcinoma progression.

SAMD5 is a protein characterized by its SAM domain, spanning approximately 70 residues and participating in diverse cellular processes through polymerization [24,25]. Although the specific functions of SAMD5 in cancers remain largely unexplored, its expression has been shown to be downregulated in primary human trabecular meshwork cells following Paired Like Homeodomain 2 (PITX2) knockdown, and it is linked to the therapeutic efficacy of chemo-radiotherapy in rectal cancer [26]. Microarray analysis by Yagai et al. [20] revealed upregulation of SAMD5 in epithelial cell adhesion molecule (EPCAM)-expressing cells from chronically injured mouse livers. IHC analysis indicated the expression of SAMD5 in human biliary epithelial cells (BECs) and cholangiocarcinoma, where it localizes to the nucleus in cancer cells and the cytoplasm in normal cells, suggesting active nuclear transport in cancer [20]. Knockdown and overexpression studies further implicated SAMD5 in regulating the cell cycle and proliferation in cholangiocarcinoma cells. Additionally, Watanabe et al. [27] reported the close association between SAMD5 expression and the therapeutic efficacy of chemoradiotherapy for rectal carcinoma. Nevertheless, its effect on breast carcinoma has not been fully elucidated. Herein, integrative bioinformatics analysis identified SAMD5 as a dramatically decreased factor in breast carcinoma tissue samples and cells. More importantly, SAMD5 is negatively correlated with c-Myc signaling, a critical signaling pathway facilitating breast cancer development [28], suggesting its potential as a tumor suppressor in breast carcinoma. As speculated, in two TNBC cell lines, SAMD5 overexpression significantly inhibited cell viability, colony formation, and cell invasion, as well as cancer biomarker levels, including Ki67, MMP2, and MMP9 [29]. These findings indicate the potential of SAMD5 as a tumor suppressor in breast carcinoma cells.

Regarding the mechanisms underlying the tumor suppressive effects of SAMD5, PLK1 has been found to be negatively correlated with SAMD5 in breast cancer. As a serine/threonine kinase family member, PLK1 plays an important role in completing the G2/M phases of the cell cycle [30,31]. As previously reported, PLK1 served as a biomarker for the prognosis of early-stage breast carcinoma [32]. More importantly, PLK1 expression was shown to be particularly increased in TNBC compared to other subtypes [32-34], and increased TNBC tumor grade (poorer differentiation and increased genome instability, leading to aggressive tumor growth) correlates with the expression level of PLK1 [32]. In this study, PLK1 knockdown indeed significantly inhibited TNBC cell viability, colony formation, and invasive capability. More importantly, PLK1 overexpression significantly attenuated the tumor-suppressive effects of SAMD5 overexpression on aggressive breast carcinoma cell phenotypes, suggesting that PLK1 mediates the functions of SAMD5 in breast cancer. In addition, PLK1 regulates mitosis activities such as centrosome disjunction, cyclin and CDK activation, spindle assembly, and chromosomal separation [35,36]. Consistently, SAMD5 overexpression significantly reduced, whereas PLK1 overexpression elevated the protein levels of key factors in the c-Myc signaling pathways. Moreover, PLK1 overexpression partially eliminated the inhibitory effects of SAMD5 on these factors, indicating that PLK1 might mediate SAMD5 functions through the c-Myc signaling. In vivo, PLK1 also partially abolished the effects of SAMD5 overexpression in inhibiting tumor growth and the c-Myc signaling in a mouse model, providing further evidence for the in vitro findings.

## Conclusions

This study identifies SAMD5 as a crucial tumor suppressor in TNBC. SAMD5 exerts its inhibitory effects on key cellular processes, including cell viability, colony formation, and invasion, while downregulating the c-Myc signaling pathway. These effects are partially mediated through its negative correlation with PLK1, a known oncogenic factor in breast cancer.

The findings presented here not only enhance our understanding of breast cancer biology but also highlight the potential of the SAMD5/PLK1 axis as a therapeutic target. Future research could be conducted to further characterize the molecular mechanisms underlying the SAMD5/PLK1 interaction and explore potential therapeutic strategies targeting this axis to improve outcomes for patients with TNBC.
